# Respiratory infections and type 1 diabetes: Potential roles in pathogenesis

**DOI:** 10.1002/rmv.2429

**Published:** 2023-02-15

**Authors:** Roy Wu, Mohsin Mumtaz, Anna J. Maxwell, Sonia R. Isaacs, Jutta E. Laiho, William D. Rawlinson, Heikki Hyöty, Maria E. Craig, Ki Wook Kim

**Affiliations:** ^1^ Discipline of Paediatrics and Child Health School of Clinical Medicine Faculty of Medicine and Health University of New South Wales Randwick New South Wales Australia; ^2^ Virology and Serology Division New South Wales Health Pathology Prince of Wales Hospital Randwick New South Wales Australia; ^3^ Department of Virology Faculty of Medicine and Health Technology Tampere University Tampere Finland; ^4^ School of Biomedical Sciences Faculty of Medicine and Health University of New South Wales Randwick New South Wales Australia; ^5^ School of Biotechnology and Biomolecular Sciences Faculty of Science University of New South Wales Randwick New South Wales Australia; ^6^ Fimlab Laboratories Tampere Finland; ^7^ Institute of Endocrinology and Diabetes Children's Hospital at Westmead Sydney New South Wales Australia; ^8^ Faculty of Medicine and Health Discipline of Child and Adolescent Health University of Sydney Sydney New South Wales Australia

**Keywords:** autoimmunity, respiratory infection, type 1 diabetes, virome, virus

## Abstract

Among the environmental factors associated with type 1 diabetes (T1D), viral infections of the gut and pancreas has been investigated most intensely, identifying enterovirus infections as the prime candidate trigger of islet autoimmunity (IA) and T1D development. However, the association between respiratory tract infections (RTI) and IA/T1D is comparatively less known. While there are significant amounts of epidemiological evidence supporting the role of respiratory infections in T1D, there remains a paucity of data characterising infectious agents at the molecular level. This gap in the literature precludes the identification of the specific infectious agents driving the association between RTI and T1D. Furthermore, the effect of severe acute respiratory syndrome coronavirus 2 (SARS‐CoV‐2) infections on the development of IA/T1D remains undeciphered. Here, we provide a comprehensive overview of the evidence to date, implicating RTIs (viral and non‐viral) as potential risk factors for IA/T1D.

**Abbreviations**
ABIS: all babies in Southeast Sweden study
CDC: centres for diseases control and prevention
CI: confidence interval
COVID‐19: coronavirus disease 2019
DIPP‐novum: type 1 diabetes prediction and prevention study
DKA: diabetes ketoacidosis
ECHO: enteric cytopathic human orphan virus
ENDIA: environmental determinants of islet autoimmunity study
EV: enterovirus
EV‐B: *Enterovirus B*
GADA: glutamic acid decarboxylase 6 antibodies
IA: islet autoimmunity
IAA: insulin autoantibodies
HLA: human leucocyte antigen
MIDIA: Norwegian environmental triggers of type 1 diabetes study
NGS: next‐generation sequencing
OR: odds ratio
*P*
: *p*‐value
RTI: respiratory tract infection
SARS‐CoV‐2: severe acute respiratory syndrome related coronavirus 2
T1D: type 1 diabetes
TEDDY: the environmental determinants of diabetes in the young study
TRIGR: Trial to reduce insulin‐dependent diabetes mellitus
VirCapSeq: virome capture sequencing

## 1 INTRODUCTION

Type 1 diabetes (T1D) is a chronic autoimmune condition affecting over nine million worldwide,^1^ characterised by the loss of functional pancreatic islet *β*‐cells. This ultimately results in the lifelong dependency on exogenous insulin.^2^, ^3^, ^4^ Although the pathophysiology of T1D is well characterised and understood, its aetiology remains unclear. However, it is well established that the mechanisms underlying the development of T1D is multifaceted and likely involves the complex interplay between genetic and environmental factors.^2^, ^5^, ^6^ Among the environmental factors associated with T1D, infections with viruses are identified as prime candidate triggers of islet autoimmunity (IA) which precedes most clinical onset of T1D.

## 2 VIRAL AETIOLOGY OF TYPE 1 DIABETES

The reduced prevalence of T1D‐associated high‐risk human leucocyte antigen (HLA) genotypes among newly diagnosed individuals, increasing global incidence of T1D,^6^, ^7^, ^8^, ^9^ seasonal variations^6^, ^10^ and geographical differences^6^, ^11^ in genetically similar individuals as well as the convergence of IA/T1D incidence of migrants to their new country of residence^12^, ^13^ all strongly support the growing contribution of environmental factors in the pathogenesis of T1D.

Several hypotheses have been proposed on how environmental factors may influence the progression of T1D. The ‘*β*‐cell overload’ hypothesis postulates that factors increasing insulin demand such as infection, growth, trauma and other physiological stresses may result in *β*‐cell dysfunction and insulin resistance, instigating and accelerating the development of IA/T1D.^6^, ^14^, ^15^, ^16^ The ‘hygiene hypothesis’ conversely states that a decrease in childhood infections due to improved hygiene may increase the incidence of autoimmune diseases like T1D.^6^, ^9^ The hygiene hypothesis proposes that a lack of childhood infections can limit immune system's exposure to various microorganisms and stunt its development, leading to an inappropriate response to future infections that may cause T1D.^9^ Another hypothesis, the ‘polio hypothesis’, suggests that the decreasing incidence of certain virus infections over time (such as enterovirus or poliovirus infections) has increased the proportion of infants who become infected in the absence of maternal antibodies that could protect against that virus, increasing the risk of complications such as *β*‐cell damage and T1D.^17^, ^18^

Among the environmental factors associated with T1D to date, viral infection has been investigated most thoroughly and hypothesised as the prime trigger of IA and progression to T1D, especially in utero and during childhood.^2^, ^13^ This is supported by a large body of molecular^6^, ^19^, ^20^ and epidemiological^21^, ^22^, ^23^, ^24^, ^25^ evidence, and multiple non‐mutually exclusive mechanisms have been proposed to explain how viral infections can induce and/or accelerate the development of IA/T1D.^26^, ^27^, ^28^, ^29^

To date, multiple viruses have been associated with T1D. Of the viruses investigated, enteroviruses (EV) have been the most deeply studied and now widely accepted as the prime candidate trigger of IA/T1D.^2^, ^29^, ^30^, ^31^ In total, over 26 different EV types have been associated with IA/T1D, mostly comprised of *Enterovirus B* (EV‐B) species members within the coxsackievirus B and enteric cytopathic human orphan virus (ECHO virus) groups.^2^, ^32^ EVs have been detected more frequently in the blood,^22^ gut^33^, ^34^ and pancreas^24^, ^35^, ^36^ of individuals with T1D compared to without, and are associated with an increased risk of T1D in prospective studies.^37^, ^38^, ^39^

## 3 RESPIRATORY TRACT INFECTIONS AND ISLET AUTOIMMUNITY/TYPE 1 DIABETES

**FIGURE 1:**
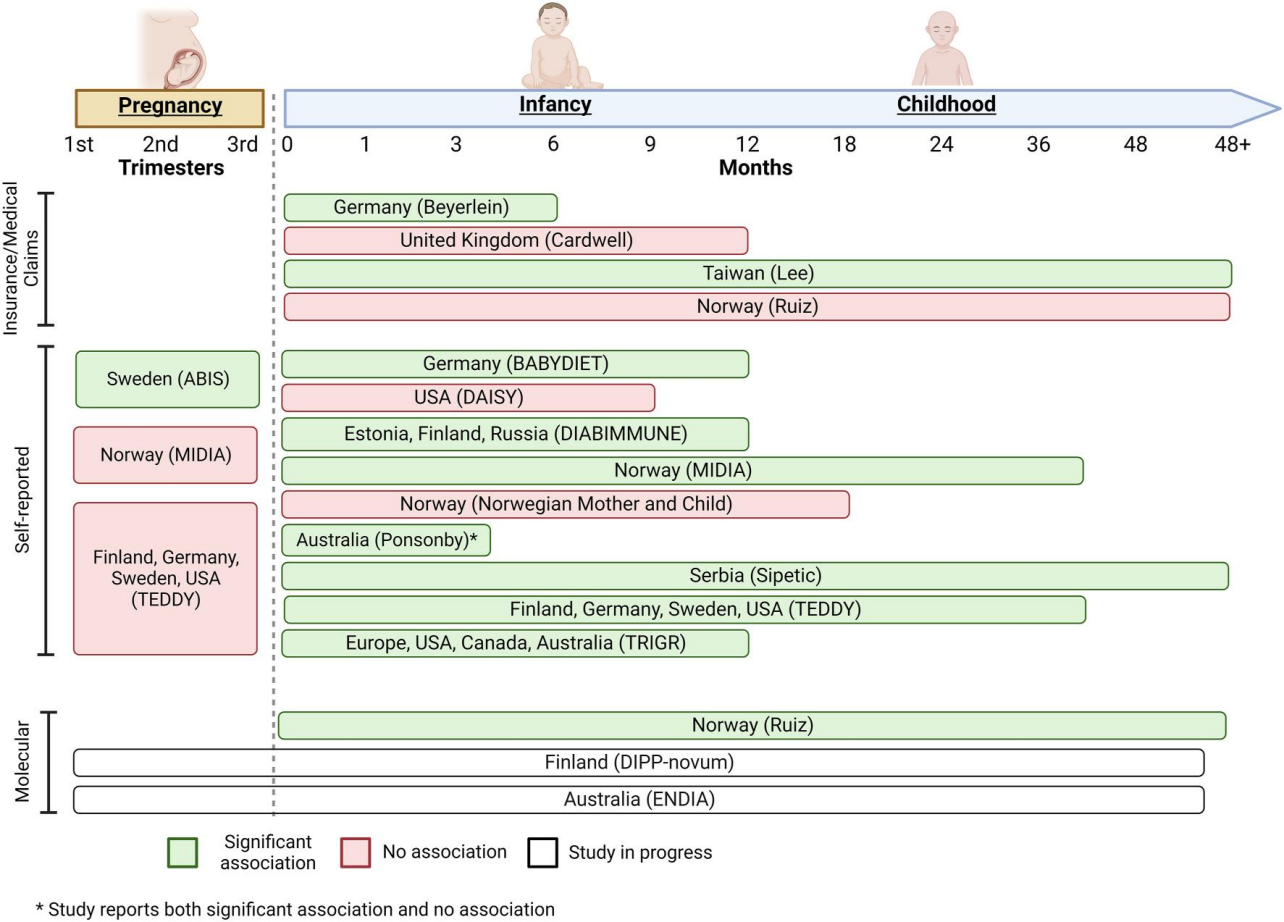
Graphic summary of studies reporting association between RTIs and islet autoimmunity (IA)/type 1 diabetes (T1D) grouped by participant age during period of infection.^26^, ^39^, ^40^, ^41^, ^42^, ^43^, ^44^, ^45^, ^46^, ^47^, ^48^, ^49^, ^50^, ^51^, ^52^, ^53^, ^59^, ^68^ Measures of exposure are categorised by insurance/medical claims data, self‐reported data and molecular data. Each study is represented by the region(s) of the study setting, with the study group or first author in brackets. ABIS = All Babies in Southeast Sweden study; DIPP = Type 1 Diabetes Prediction and Prevention study; MIDIA = Norwegian Environmental Triggers of Type 1 Diabetes study; TEDDY = the Environmental Determinants of Diabetes in the Young study; TRIGR = Trial to Reduce Insulin‐Dependent Diabetes Mellitus.

Although most research to date on the infectious aetiology of IA/T1D have focussed heavily on viral infections in the gut and pancreas,^2^, ^5^, ^30^ respiratory tract infections (RTI), particularly within the first 12 months after birth,^26^, ^40^, ^41^, ^42^ have also been investigated as a potential risk factor for childhood T1D. Both lower RTIs (including pneumonia, bronchitis and bronchiolitis) and upper RTIs (including rhinitis, pharyngitis and laryngitis) have been examined by at least 19 observational studies as potential triggers for IA/T1D development (Figure 1, Table 1).

**TABLE 1:** Studies that investigated the relationship between respiratory tract infections (RTI) and islet autoimmunity (IA)/type 1 diabetes (T1D).

Study (year/region)	Cases (controls)	Outcome	Exposure	OR (95% CI)	*p*‐value
Insurance/Medical claims					
Beyerlein (2016/Germany)^26^	720 (294424)	T1D	≥1 RTI in the first 6 months of life	1.17 (1.00‐1.37)	<0.05
Cardwell (2008/United Kingdom)^45^	367 (4579)	T1D	Medical consultations in first year of life for		
			Upper RTI	0.84 (0.67‐1.04)	0.11
			Lower RTI	0.81 (0.55‐1.20)	0.28
Lee (2015/Taiwan)^43^	632 (6320)	T1D	≥1 acute RTI	1.74 (1.30‐2.33)	<0.05
			≥1 episode of pneumonia or influenza	1.80 (1.35‐2.41)	<0.05
Ruiz (2018/Norway)^44^	2376 (2284274)	T1D	Pandemic influenza infection	1.19 (0.97‐1.46)	>0.05
			Laboratory confirmed pandemic influenza infection	2.26 (1.51‐3.38)	<0.05
Self‐reported (postnatal)					
BABYDIET (2013/Germany)^40^	26 (122)	IA	RTI in the first 6 months of life	2.27 (1.32‐3.91)	<0.05
			RTI in 6–12 months of life	1.32 (1.08‐1.61)	<0.05
			Upper RTI in 6 months prior to seroconversion	1.57 (1.26‐1.95)	<0.05
			Lower RTI in 6 months prior to seroconversion	1.28 (0.51‐3.17)	>0.05
DAISY (2012/USA)^50^	109 (1620)	IA	Upper respiratory symptoms (cough, cold, runny nose, stuffy nose, sinus infection, ear infection) in first 9 months of life	1.00 (0.98‐1.01)	0.65
			Respiratory disease (croup, pneumonia, bronchitis) in first 9 months of life	0.99 (0.67‐1.74)	0.98
Diabimmune (2018/Estonia, Finland, Russia)^41^	46 (744)	IA & T1D	Number of respiratory infections per child in the first year of life (IA cases vs. controls)		0.003
			Number of respiratory infections per child in the first year of life (T1D cases vs. controls)		0.002
MIDIA (2011/Norway)^46^	42 (843)	IA	≥1 lower RTI by 4 years of life	3.4 (1.6‐7.1)	0.001
Norwegian Mother and child (2018/Norway)^49^	286 (70154)	IA	Upper RTI in first 18 months of life		
			0‐3	1.00 (reference)	
			4‐5	0.97 (0.69‐1.38)	0.88
			6‐7	0.99 (0.69‐1.42)	0.97
			≥8	1.96 (0.77‐1.45)	0.50
			≥1 lower RTI in first 18 months of life	0.85 (0.59‐1.21)	0.36
Ponsonby (2011/Australia)^42^	26 (10602)	T1D	≥1 upper RTI by 5 weeks of life	2.74 (1.19‐6.32)	0.02
			≥1 upper RTI by 12 weeks of life	1.55 (0.65‐3.69)	0.32
Sipetic (2003/Serbia)^59^	105 (210)	T1D	Frequent (≥3 infections per year) RTI	2.65 (1.37‐5.11)	<0.01
TEDDY (2003/USA)^52^	52 (1210)	IA	≥1 episode of RTI symptoms during pregnancy	0.66 (0.38‐1.15)	>0.05
TEDDY (2017/Finland, Germany, Sweden, USA)^47^	454 (7415)	IA	Respiratory infectious episodes during winter	1.43 (1.17‐1.75)	0.0005
			Common cold	1.38 (1.11‐1.71)	0.004
			Influenza‐like illness	2.37 (1.35‐4.15)	0.003
			Sinusitis	2.63 (1.22‐5.67)	0.01
			Laryngitis/tracheitis	1.76 (1.04‐2.98)	0.04
TRIGR (2022/Europe, USA, Canada, Australia)^48^	842 (1175)	IA & T1D	Upper RTI in first 12 months of life (IA as outcome)	1.20 (1.00‐1.44)	0.044
			Upper RTI in first 12 months of life (T1D as outcome)	1.05 (0.73‐1.50)	0.797
Self‐reported (pregnancy)					
ABIS (2022/Sweden)^53^	137 (16155)	T1D	≥1 RTI during pregnancy	1.49 (1.01‐2.22)	0.04
			≥1 RTI during first trimester	2.31 (1.32‐4.04)	0.002
			≥1 RTI during second trimester	1.10 (0.59‐2.04)	0.77
			≥1 RTI during third trimester	1.15 (0.56‐2.35)	0.71
MIDIA (2011/Norway)^46^	42 (843)	IA	RTI during pregnancy		
			1	1.23 (0.96‐1.58)	0.09
			≥2	0.98 (0.74‐1.30)	0.87
TEDDY (2018/Finland, Germany, Sweden, USA)^51^	438 (7034)	IA	Gestational RTI (IAA as outcome)	0.88 (0.67‐1.15)	0.35
			Gestational RTI (GADA as outcome)	0.95 (0.73‐1.25)	0.73
Molecular					
Ruiz (2018/Norway)^44^	2376 (2284274)	T1D	Laboratory confirmed pandemic influenza infection	2.26 (1.51‐3.38)	<0.05

Abbreviations: ABIS, All Babies in Southeast Sweden study; CI, confidence interval; GADA, glutamic acid decarboxylase 6 antibodies; IA, islet autoimmunity; IAA, insulin autoantibodies; MIDIA, Norwegian Environmental Triggers of Type 1 Diabetes study; OR, odds ratio; RTI, respiratory tract infection; T1D, type 1 diabetes; TEDDY, the Environmental Determinants of Diabetes in the Young study; TRIGR, Trial to Reduce Insulin‐Dependent Diabetes Mellitus.

Three retrospective case‐control and cohort studies reported a significant association between RTIs and T1D,^26^, ^43^, ^44^ while two reported no association.^44^, ^45^ Limited sampling methods and heterogeneity in study design between studies may have contributed to inconsistent results. These studies relied on insurance claims or medical consultation data to ascertain RTI exposure, which only capture clinically overt symptomatic infections. Hence, such studies are likely to have underestimated the cumulative exposure to RTIs. Only one retrospective study included molecular testing to confirm the infectious agent, reporting a significant association between laboratory confirmed pandemic influenza A (H1N1) and T1D, but not between clinically diagnosed H1N1 and T1D.^44^ All these studies lacked IA testing, precluding the examination of IA as an outcome associated with RTIs.

Prospective birth cohort studies investigating IA as an outcome have reported that early‐life RTIs increased the risk of IA.^40^, ^41^, ^46^, ^47^, ^48^ These studies followed genetically at‐risk children from birth (as determined by HLA genotype and/or family history of T1D), prospectively collecting data on RTIs through questionnaires and health event logs, and performing regular blood tests to monitor the timing of seroconversion to IA. Norwegian and German studies reported a higher prevalence of IA in children with ≥1 RTI in the first 4 years of life [odds ratio (OR) 3.4, 95% confidence interval (CI) 1.6–7.1, *p*‐value (*p*) = 0.001]^46^ and first 6 months of life (OR 2.27, 95% CI 1.32–3.91, *p* < 0.05).^40^ These findings were supported by two large‐scale American/European birth cohort studies, the Environmental Determinants of Diabetes in the Young, which reported the risk of IA increased by 5.6% for every RTI recorded in children up to 4 years of age,^47^ and the Trial to Reduce Insulin‐Dependent Diabetes Mellitus, which reported that upper RTIs in the first 12 months of life was associated with IA (OR 1.20, 95% CI 1.00–1.44, *p* = 0.04).^48^ In contrast, other large European^49^ and American^50^ studies found no significant association between early‐life RTIs and IA. These conflicting results may be partly due to the limitations of analysing subjective data types, necessitating further research using molecular methods to definitively confirm and characterise infections and any viruses causing these infections.

Viral exposures in utero have been hypothesised as possible causes of IA/T1D. While most studies did not find an association between gestational RTI and IA/T1D,^46^, ^51^, ^52^ a recent report from the All Babies in Southeast Sweden Study showed that gestational RTIs during the first trimester were associated with higher risk of T1D in offspring (OR 2.31, CI 1.32–4.04, *p* = 0.002).^53^ A plausible explanation is that since the first trimester coincides with the embryological development of the pancreas, a congenital infection during early pregnancy may prime the offspring's immune system and pancreas to produce islet autoantibodies during a second infection postnatally, whereas a more developed pancreas would be less susceptible.^53^ However, as no other studies have replicated these results, external validation in other prospective cohorts with maternal data and respiratory samples collected longitudinally during pregnancy is needed.

Specific respiratory viruses including parechoviruses and influenza virus have been associated with T1D in retrospective studies and animal studies. One mouse study found an association between a strain of parechovirus (Ljungan virus) and T1D.^54^ While one Japanese retrospective cohort study reported an increased risk of T1D after the diagnosis of influenza,^55^ and an Italian study found increased incidence of T1D diagnoses during the 2009 H1N1 pandemic,^56^ most observational studies did not find an association between influenza^57^, ^58^, ^59^, ^60^ or parechoviruses^61^ and T1D in humans. In addition, many EV species replicate in the respiratory tract, and the most common manifestation of EV infection is a common cold‐type disease. These EV species include rhinoviruses which are responsible for over 50% of all RTIs,^2^ EV‐B,^62^ and members belonging to *Enterovirus C* ^63^ and *D*,^64^ that replicate primarily in the respiratory tract. Despite this, no epidemiological studies have examined EVs from respiratory samples in the context of IA/T1D.

The lack of molecular data in most retrospective and prospective studies is a key limitation to the identification of specific infectious agents (viral or non‐viral) that may be driving the association between RTIs and T1D. Molecular characterisation of infectious agents using comprehensive next‐generation sequencing (NGS) methods such as virome capture sequencing (VirCapSeq) can overcome this limitation by enabling sensitive characterisation of all viruses in a given specimen, with minimal investigation bias.^2^, ^7^ Despite this, there remains no comprehensive molecular study to date that has investigated the respiratory virome in at‐risk individuals.^65^, ^66^, ^67^ Hence, large‐scale molecular research involving NGS that focuses on the association between RTI and IA/T1D is needed to support existing epidemiological studies. Current birth cohort studies including the Environmental Determinants of Islet Autoimmunity (ENDIA)^68^ and Diabetes Prediction and Prevention novum (DIPP‐novum)^69^ study are in progress that prospectively follow participants from in utero throughout childhood with molecular testing of the respiratory virome, which may shed further information on the relationship between RTI and T1D.

Recently, a machine learning approach was used to rank tissue‐specific transcription regulatory effects for single‐nucleotide polymorphisms in T1D associated genes, estimating their relative contributions to the development of T1D by integrating T1D case and autoantibody‐negative control genotypes with tissue‐specific quantitative trait loci (eQTL) data.^70^ The investigators found that the largest gene regulatory contribution to the risk of T1D development was made by the rs6679677 eQTL, which is associated with changes to *AP4B1‐AS1* transcript levels in lung tissues. Therefore, the strongest tissue‐specific eQTL effects associated with T1D risk occurred in the lung, supporting the potential contribution of respiratory infections on the development of IA/T1D.

## 4 CORONAVIRUS INFECTION AND TYPE 1 DIABETES

Severe acute respiratory syndrome related coronavirus 2 (SARS‐CoV‐2) infection and its related disease, coronavirus disease (COVID‐19), has an unclear relationship with T1D. Although several recent studies have reported possible associations between SARS‐CoV‐2 infection and IA/T1D,^71^, ^72^, ^73^, ^74^ it remains too early to draw any meaningful conclusions. Like other viruses, SARS‐CoV‐2 infections can induce a stress response that may diminish insulin secretion, release counter‐regulatory hormones like cortisol and adrenaline, induce excessive gluconeogenesis and impair glucose disposal, thereby causing transient hyperglycaemia. However, these mechanisms may not necessarily cause diabetes.^75^, ^76^, ^77^

The mechanism of how SARS‐CoV‐2 may cause T1D has been explored within in vitro and ex vivo studies. The detection of SARS‐CoV‐2 in post‐mortem pancreatic samples^78^, ^79^, ^80^ and reduced pancreatic function in people with COVID‐19^81^ suggests SARS‐CoV‐2 and its related virus SARS‐CoV‐1 may damage pancreatic *β*‐cell and cause new‐onset diabetes via direct infection and the subsequent inflammatory response and interactions with the renin‐angiotensin system.^81^, ^82^, ^83^, ^84^, ^85^, ^86^, ^87^, ^88^ Nevertheless, whether the infection of pancreatic *β*‐cells in tissue samples accurately mimics in vivo infection remains unclear.

Studies investigating associations between SARS‐CoV‐2 and T1D have been steadily increasing across the last 3 years. Cross‐sectional studies^82^, ^83^, ^84^, ^85^, ^86^, ^87^ have reported an increase in incidence of T1D and diabetic ketoacidosis (DKA) during the pandemic, and there are case reports^89^, ^90^, ^91^, ^92^, ^93^, ^94^ of individuals with recent SARS‐CoV‐2 infection presenting to hospital with new‐onset T1D and DKA, which suggest that SARS‐CoV‐2 infection may accelerate T1D development or increase the risk of its metabolic complications. However, the increased incidence of DKA and T1D during the pandemic may be confounded by reduced access or hesitancy to use healthcare services, leading to delayed presentations of T1D and higher incidence of DKA,^95^, ^96^ and individuals presenting to hospital with COVID‐19 may have pre‐existing undiagnosed T1D.

National retrospective cohort studies based on medical claims databases have reported mixed results regarding the incidence of T1D following SARS‐CoV‐2 infections. A US Centres for Disease Control and Prevention (CDC) paper^97^ using two US medical claims databases reported a significantly higher risk of new‐onset diabetes 30 days or more after SARS‐CoV‐2 infection in persons under 18 years. While the CDC report included all types of diabetes which lowers specificity, another national retrospective cohort in the US found higher risk of new‐onset T1D and DKA in individuals with previous SARS‐CoV‐2 infection.^98^ A similar retrospective cohort in Scotland also reported an association between SARS‐CoV‐2 infection and T1D, but only for infection within the 30 days of T1D onset.^99^ It is plausible that SARS‐CoV‐2 infection may acutely contribute to the accelerated progression of symptomatic T1D and diagnosis in at risk individuals, which aligns with the role of other viruses, such as enteroviruses, in the progression to clinical T1D.^100^ However, since transient hyperglycaemia is associated with SARS‐CoV‐2 infection,^76^ T1D may have been misdiagnosed during the acute stages of SARS‐CoV‐2 infection. Furthermore, higher opportunistic testing rates around the time of presentation of either SARS‐CoV‐2 or T1D may have also contributed to incidental diagnosis of the secondary condition, and SARS‐CoV‐2 may trigger metabolic decompensation that precipitates diagnosis of nascent T1D,^97^, ^99^ limiting the strength of these associations.

A meta‐analysis of eight retrospective cohort studies comprising 3700 hospitalised COVID‐19 patients found 14.4% had new‐onset T1D.^101^ However, the meta‐analysis^101^ of retrospective studies included individuals ranging from 47.0 to 64.9 years in age, outside of the typical age range when T1D is diagnosed, which may suggest an alternative pathogenesis. Indeed, several case reports feature individuals with new‐onset autoantibody‐negative T1D on a background of COVID‐19.^90^, ^93^ A prospective study that followed people with DKA and autoantibody‐negative T1D after COVID‐19 reported that most individuals achieved *β*‐cell recovery and insulin independence, suggesting an autoantibody‐negative T1D in contrast with the IA pathway classically seen in T1D.^102^ Nevertheless, additional mechanistic studies are needed to validate this pathogenic hypothesis.

The relationship between COVID‐19 and T1D remains a poorly understood and rapidly evolving area of research, with its long‐term diabetogenic effects likely to be unknown until after many years of extensive research. To this end, a global registry (CoviDiab) was established to investigate their interaction.^103^ Long‐term prospective analysis is needed to decipher any relationship between COVID‐19 and T1D.

## 5 CONCLUSION

There is an enormous body of accumulated evidence, both molecular and epidemiological, that support the hypothesised role of viral infections in the development of IA and T1D. By comparison, there remains a major gap in understanding and paucity of data, especially molecular data where infectious agents are characterised at the nucleic acid or protein level, that elucidates the relationship between RTI and IA/T1D. To address this gap, the use of comprehensive metage detection methods, and the prospective collection of respiratory samples and IA testing during pregnancy and early life in large prospective cohorts such as the ENDIA,^68^ TEDDY^47^ and DIPP‐novum^39^ will be important. If a clinically significant association between specific respiratory viruses and T1D are established in the future, primary prevention of T1D may be possible through antiviral vaccines.

## AUTHOR CONTRIBUTIONS

**Roy Wu**: writing—original draft; editing. **Mohsin Mumtaz**, **Anna J. Maxwell**, **Sonia R. Isaacs**, **Jutta E. Laiho**, **William D. Rawlinson**, **Heikki Hyöty**: reviewing, editing. **Maria E. Craig**, **Ki Wook Kim**: conceptualisation; preparation; writing; editing.

## CONFLICT OF INTEREST STATEMENT

None of the authors have any conflicts of interest to declare.

## Data Availability

All data used are available in this review.
